# Zinc-Doped Iron Oxide Nanoparticles as a Proton-Activatable Agent for Dose Range Verification in Proton Therapy

**DOI:** 10.3390/molecules28196874

**Published:** 2023-09-29

**Authors:** Marta Ibáñez-Moragues, Irene Fernández-Barahona, Rocío Santacruz, Marta Oteo, Víctor M. Luján-Rodríguez, María Muñoz-Hernando, Natalia Magro, Juan I. Lagares, Eduardo Romero, Samuel España, Andrea Espinosa-Rodríguez, Miguel García-Díez, Víctor Martínez-Nouvilas, Víctor Sánchez-Tembleque, José Manuel Udías, Víctor Valladolid-Onecha, Miguel Á. Martín-Rey, Edilia I. Almeida-Cordon, Sílvia Viñals i Onsès, José Manuel Pérez, Luis Mario Fraile, Fernando Herranz, Miguel Ángel Morcillo

**Affiliations:** 1 Centro de Investigaciones Energéticas, Medioambientales y Tecnológicas CIEMAT, Medical Applications of Ionizing Radiation Unit, 28040 Madrid, Spain; rociosantacruzsampalo@gmail.com (R.S.); marta.oteo@ciemat.es (M.O.); victormanuel.lujan@ciemat.es (V.M.L.-R.); natalia.magro@ciemat.es (N.M.); juanignacio.lagares@ciemat.es (J.I.L.); eduardo.romero@ciemat.es (E.R.); jm.perez@ciemat.es (J.M.P.); 2 Facultad de Farmacia, Universidad Complutense de Madrid, 28040 Madrid, Spain; irenefbarahona@gmail.com; 3 Instituto de Química Médica—Consejo Superior de Investigaciones Científicas IQM-CSIC, Nanomedicine and Molecular Imaging Group, 28006 Madrid, Spain; mmunozhernando@gmail.com (M.M.-H.);; 4 Nuclear Physics Group, Universidad Complutense de Madrid, IPARCOS &EMFTEL, CEI Moncloa, 28040 Madrid, Spain; sespana@ucm.es (S.E.); anespi04@ucm.es (A.E.-R.); miguga20@ucm.es (M.G.-D.); victor.m.nouvilas@ucm.es (V.M.-N.); victor.sanchez-tembleque@hesge.ch (V.S.-T.); jmudiasm@ucm.es (J.M.U.); vicvalla@ucm.es (V.V.-O.); lmfraile@ucm.es (L.M.F.); 5 Instituto de Investigación del Hospital Clínico San Carlos (IdISSC), Ciudad Universitaria, 28040 Madrid, Spain; 6 Centro de Investigaciones Energéticas, Medioambientales y Tecnológicas CIEMAT, Hematopoietic Innovative Therapies Unit, 28040 Madrid, Spain; miguelangel.martin@ciemat.es; 7 Centro de Investigaciones Energéticas, Medioambientales y Tecnológicas CIEMAT, Animal Facility Unit, 28040 Madrid, Spain; edilia.almeida@ciemat.es; 8 Center for Microanalysis of Materials (CMAM), Universidad Autónoma de Madrid, 28049 Madrid, Spain; silvia.vinnals@uam.es

**Keywords:** radiotherapy, nanoparticle, proton range verification, proton therapy, iron oxide nanoparticles, zinc, irradiation, prompt gamma radiation

## Abstract

Proton therapy allows the treatment of specific areas and avoids the surrounding tissues. However, this technique has uncertainties in terms of the distal dose fall-off. A promising approach to studying the proton range is the use of nanoparticles as proton-activatable agents that produce detectable signals. For this, we developed an iron oxide nanoparticle doped with Zn (IONP@Zn-cit) with a hydrodynamic size of 10 nm and stability in serum. Cytotoxicity, defined as half of the surveillance, was 100 μg Zn/mL in the U251 cell line. The effect on clonogenic cell death was tested after X-ray irradiation, which suggested a radioprotective effect of these nanoparticles at low concentrations (1–10 μg Zn/mL). To evaluate the production of positron emitters and prompt-gamma signals, IONP@Zn-cit was irradiated with protons, obtaining prompt-gamma signals at the lowest measured concentration (10 mg Zn/mL). Finally, ^67^Ga-IONP@Zn-cit showed accumulation in the liver and spleen and an accumulation in the tumor tissue of 0.95% ID/g in a mouse model of U251 cells. These results suggest the possibility of using Zn nanoparticles as proton-activatable agents to verify the range by prompt gamma detection and face the challenges of prompt gamma detection in a specific biological situation, opening different avenues to go forward in this field.

## 1. Introduction

Proton-beam therapy has attracted interest because of the unique properties of protons. Protons and charged particles generally stop at specific depths, depending on the energy and density of the irradiated material. They exhibit a relatively low ionization density at the surface or entrance of the material, which increases slowly until near the end of the beam, when there is high-dose deposition in a small area of the material known as the Bragg peak. In the context of cancer treatment, this characteristic dose deposition enables the use of protons to increase the dose in tumor tissue, producing different effects at the molecular level compared with conventional radiotherapy [1], and significantly avoiding undesirable doses in critical organs and surrounding healthy tissues [2,3,4].

However, the large-scale clinical use of proton beam precision is hampered by uncertainties in the location of the distal dose fall-off in the patient’s body. Proton treatment-planning strategies often only utilize the lateral penumbra of the beam in the proximity of critical organs for dose conformation [5]. To understand the true uncertainties, reduce delivery errors, and improve dose escalation, in vivo verification of the delivered dose or range is highly desirable [4,6]. Different methods can be proposed and classified depending on the measurement technique (direct or indirect), timing (online or offline), and dimension (1D, 2D, or 3D) [4]. The obvious choice is the use of in vivo dosimetry detectors that would allow real-time dose reporting. However, this approach is only feasible for very few indications, such as prostate cancer, in which detectors can be placed on the rectal balloon used for immobilization. A promising approach is the use of imaging methods. The use of imaging devices to monitor treatment delivery is common practice in conventional photon therapy, where each beam penetrates the patient and the exit dose can be measured. By contrast, protons stop inside the patient; thus, imaging can only be based on the secondary radiation generated by the primary beam. In this context, several methods have been proposed for range verification in proton therapy, such as in vivo point dose measurements, implanted markers, proton radiography, and tomography, or indirect methods such as prompt gamma [7], PET, MRI, ultrasound imaging [4,8], and ion-acoustic measurements [9], among others. Depending on the case, imaging can be performed either “online”, i.e., during the treatment, or “offline”, i.e., after the treatment has been completed [10].

Two of the most promising methods for online verification are based on in-elastic collisions, in which protons undergo nuclear changes in the tissues [3,6,10]. The first is based on the detection of positron-emitting isotopes generated by the nuclear interactions of the proton beam in tissues that can be detected by positron emission tomography (PET). The idea of using PET for proton range verification dates back to the 1970s. The positron emitters of main interest are ^11^C and ^15^O, with half-lives of approximately 20 and 2 min, respectively. However, these isotopes are produced by nuclear interactions along the proton beam path with a relatively high energy production threshold, implying that they will not be formed near the Bragg peak [11]. As a result, an alternative methodology has been proposed that consists of using contrast agents [12,13,14,15,16,17] and external media that facilitate the production of detectable activity closer to the Bragg peak. One of the proposed materials is zinc, which leads to the production of isotopes such as ^66^Ga and ^68^Ga via proton-induced reactions [12]. This may allow for better measurement of the dose distribution deposited by protons at the distal end of the trajectory.

In this study, we examined the possible role of zinc in the form of nanoparticles as a contrast agent for the target volume, as the gallium isotopes produced during the proton therapy treatment from the natZn(p,x)Ga reactions in the zinc material may then be measured after irradiation. The combination of the information from the nanoparticles provided by MR and/or PET images, together with an accurate knowledge of the cross-section of these reactions, would allow precise measurement of the dose deposited by low-energy protons [12]. Moreover, measurements performed offline have the advantage that it is not necessary for the PET detector to be in the same room where the treatment is carried out, but the problem is the rapid decrease that some radioisotope signals could have owing to decay and biological washout. Images taken offline predominantly show activity from radioisotopes, whose half-life is longer than the time it takes to transfer the patient to another facility where the PET scanner is placed [5,10].

The second promising method is based on ray-gamma detection during proton irradiation of tissues, showing an alternative approach for in vivo range verification using prompt γ-rays (PG). PGs are photons emitted promptly (~1 ns after proton irradiation of the material) during the de-excitation of nuclear reaction products, resulting from collisions between protons and nuclei of the medium along the beam path [18]. The emission of PGs is non-isotropic, with discrete gamma characteristic peaks that correspond to unique gamma de-excitations of nuclear states with an energy below 10 MeV. The PG distribution has been correlated with the distal dose fall-off of a proton beam incident in a water tank and, thus, with its range [18]. The latest technique was first introduced in 2003 by Stichelbaut and Jongen [19], and the first clinical prototype for PG range verification during proton therapy was developed by Gonzalez et al. [20]. It offers distinct advantages compared to PET because of the much higher emission count rate and lack of biological washout. In combination with a high-efficiency detector, the expected count rate theoretically allows real-time range verification. Another advantage is that the maximum in the nuclear interaction cross-section, leading to prompt γ-rays, appears at a lower energy compared to the production of positron emitters [6,10,21]. Despite encouraging preliminary results from several research groups, verification methods still pose challenges [18].

The relevance of nanomaterials in science and technology has significantly increased in recent years, and technological advances in nanoparticle synthesis and functionalization have triggered significant progress in molecular detection and imaging, targeting, and multifunctional therapeutics [22]. The increased surface-to-volume ratio and reactivity of nanomaterials facilitate their use in diagnostics, therapeutics, drug delivery systems, electronics, cosmetics, personal care products, and food additives because of their magnetic, catalytic, semiconducting, antimicrobial, and ultraviolet-protective properties, which are size dependent [23]. Nanoparticles (NPs) are defined by a particle size of less than 100 nm [24,25]. Iron oxide nanoparticles (IONPs) have been extensively used as imaging probes. Their superparamagnetic behavior, biocompatibility, and chemical stability make them particularly important NPs for biomedical applications, such as their use as hybrid probes for multimodal PET/MRI imaging [26]. Moreover, nanomaterials can be directed to tumors either by the enhanced permeability and retention effect (EPR) or by using targeting moieties [26,27]. To provide signals in PET, IONPs have been radiolabeled with different radioisotopes such as ^64^Cu, ^68^Ga, ^18^F, ^124^I, ^11^C, and ^89^Zr [26]. Some studies have demonstrated that metal-based nanoparticles injected into tumors amplify proton radiation treatment efficiency and increase the mean tissue density in the planning tumor volume (PTV), resulting in a higher proton stopping power when the charged particle crosses the tissue. Although this hypothesis has been largely studied in terms of the radiosensitization mechanism to improve the biological effects of the physical irradiation dose [28,29], the opportunity to use them as PET or PG signal enhancers remains poorly investigated [30], and few studies have been conducted from a biological perspective.

The main objective of our study was to assess the feasibility of an in vivo proton-range verification method based on iron oxide nanoparticles doped with Zn, named IONP@Zn-cit, as an activatable agent to improve PET and PG detection. We modified previously developed iron oxide nanoparticles (IONPs) [31], which are useful for PET imaging owing to radioisotope labeling or themselves for MRI imaging and doped them with Zn. This opens a window not only for their use as proton-activatable agents but also for future modifications to use them in other emerging fields (e.g., other imaging techniques, targeting, and treatments [32]). Cytotoxicity, nanoparticle effect on cell viability under radiation, production of PET and prompt-gamma signals after proton irradiation, and biodistribution in an animal model were studied in this work.

## 2. Results

### 2.1. Nanoparticle Synthesis and Characterization

**Figure 1:**
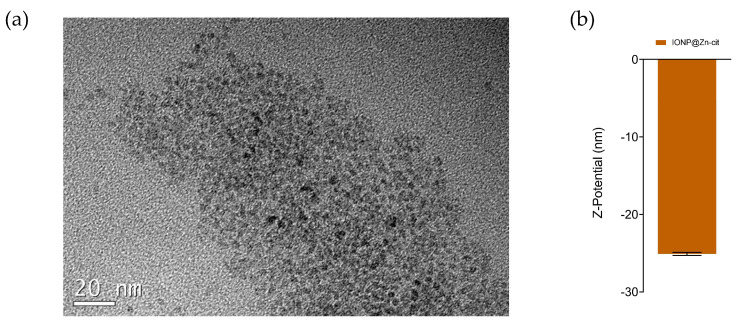
(**a**) TEM image of IONP@Zn-cit (scale bar: 20 nm); (**b**) zeta potential of IONP@Zn-cit.

IONP@Zn-cit was synthesized for in vitro studies using a microwave-assisted protocol that enables highly homogeneous and reproducible synthesis in a very short reaction time. We obtained nanoparticles with extremely small cores of around 3 nm as observed by electron microscopy (TEM) (Figure 1a) and a 10 nm mean hydrodynamic size with a narrow size distribution measured by dynamic light scattering. Their zeta potential was −25.10 ± 0.20 nm (Figure 1b), as expected for citrate-coated nanoparticles. This citrate confers colloidal stability, biocompatibility, and hydrophilic behavior to the NP [33]. The metal composition was studied by ICP-MS, which yielded a core composition of 60.50 ± 0.05% Zn and 39.50 ± 0.05% Fe.

Additionally, NP colloidal stability in cell culture medium was tested by incubating different concentrations of IONP@Zn-cit in cell culture medium with and without 10% fetal bovine serum (FBS) and checked visually after 3 h and 7 days. The nanoparticles in DMEM without 10% FBS supplementation were unstable and tended to aggregate after only 3 h of incubation (Figure S1). In contrast, nanoparticles incubated in DMEM supplemented with 10% FBS did not undergo aggregation, even after 7 days of incubation, suggesting that serum proteins would promote colloidal stability. In view of these results, all in vitro experiments were performed using supplemented cell culture medium.

For radiolabeled nanoparticles, a core-doping, chelator-free approach was used [34]. In the ^67^Ga-IONP@Zn-cit synthetic process, we collected the fractions and measured their activities to obtain a high radiolabeling yield (77%) (Figure S2). Metal concentrations were checked by ICP-MS measurements, resulting in 1.06 mg Zn/mL and 0.69 mg Fe/mL. 

### 2.2. IONP@Zn-cit Cytotoxicity

**Figure 2:**
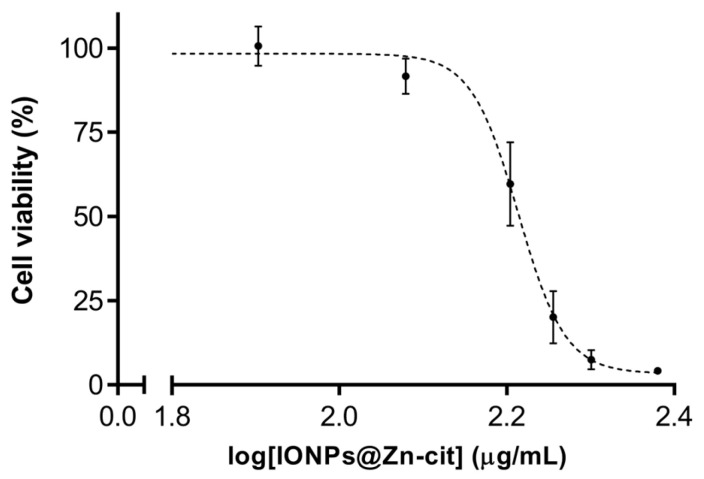
Sigmoidal adjustment was based on the results of the MTT assay when U251 cells were incubated with different amounts of IONP@Zn-cit.

Nanoparticle cytotoxicity was tested in a human malignant glioblastoma multiforme cell line (U251) using the 2,5-diphenyl-2H-tetrazolium bromide (MTT) assay. The percentage of surveillance data adjusted to a sigmoidal curve is shown in Figure 2. IONPs@Zn-cit showed a dose-dependent cytotoxic effect after 24 h of exposure, with IC50 values of 64 µg Fe/mL and 100 µg Zn/mL (Table 1). Considering these data and other nanoparticle studies [27,35,36,37], non-toxic concentrations for in vitro studies (1 and 10 µg Zn/mL) and a low dosage for the in vivo biodistribution study were used.

### 2.3. Influence of IONP@Zn-cit on X-ray Radiation-Induced Clonogenic Cell Death 

**Figure 3:**
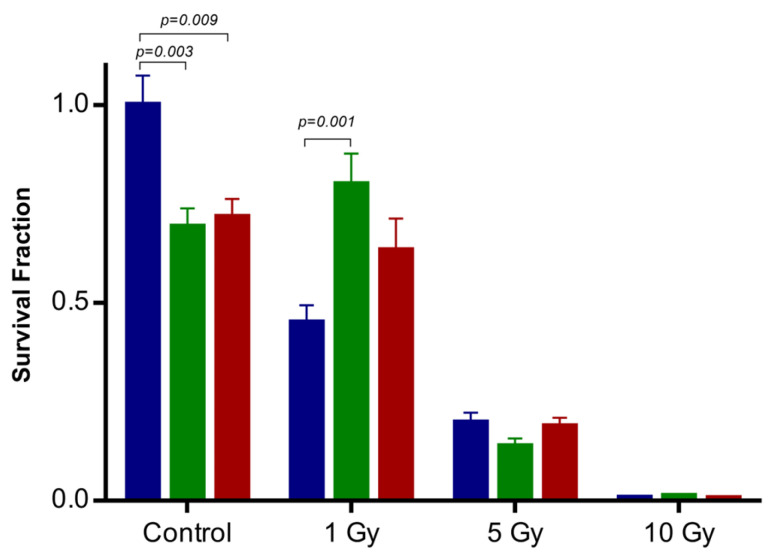
Clonogenic cell survival curve following X-irradiation for each IONP@Zn-cit concentration: without (blue) or with NPs containing 1 (green) or 10 µg (red) Zn/mL. Data are expressed as mean ± SEM.

The radiobiological impact of IONP@Zn-cit on the U251 cell line exposed to different doses of 250 kV X-rays was assessed using a clonogenic assay. Considering the possible physical, chemical, and biological mechanisms that can influence the NP radiation–enhancement effect, we selected non-toxic concentrations to test only the effect of NPs on the effectiveness of radiation. For this purpose, cells were incubated for 24 h prior to irradiation with IONP@Zn-cit nanoparticle concentrations of 1 or 10 µg Zn/mL. To further examine the radio-sensitization/protection effect of IONP@Zn-cit NPs, a clonogenic assay was performed. As it is shown in Figure 3, the cell surviving fraction (SF) decreased in the presence of NPs, without X-irradiation, to 0.69 ± 0.30 (1 µg Zn/mL) and 0.72 ± 0.29 (10 µg Zn/mL), with differences statistically significant (*p* = 0.003 and *p* = 0.009, respectively). SF decreased with increasing doses of X-ray radiation (in the absence or presence of NPs), with the exception of NP-treated U251 cells (1 µg Zn/mL) under 1 Gy X-ray radiation, where SF was significantly higher than that of non-treated cells (0.45 ± 0.22 vs. 0.80 ± 0.38, *p* = 0.001). The SF in NP-treated U251 cells (10 µg Zn/mL) under 1 Gy X-ray radiation also increased, although the difference was not statistically significant (0.45 ± 0.22 vs. 0.63 ± 0.40, *p* = 0.149). 

Table 2 lists the estimated parameters from the survival curves fitted to the linear quadratic model (LQM). The α parameter decreased in the presence of nanoparticles compared to the ionizing radiation alone group, whereas the β parameter exhibited a reverse tendency and decreased. All biological endpoints (SF_2Gy_, D_50%_, D_10%_, and MID) increased in the presence of nanoparticles compared with the group treated alone with ionizing radiation. Additionally, the DEF_2Gy_ value was used to evaluate the effect of the NPs in a well-defined scenario (2 Gy, a clinically relevant radiation dose). The DEF_2Gy_ mean value was 0.490 in the combined groups (NP + X-ray); therefore, this finding indicated that it was necessary to induce a 2-fold dose in the presence of NPs to induce the same survival fraction of cells obtained without nanoparticles at 2 Gy. Overall, these results revealed that IONPs@Zn-cit nanoparticles have a significant radioprotective effect on U251 glioblastoma cancer cells.

### 2.4. Proton Irradiation of IONP@Zn-cit

To assess the feasibility of in vivo monitoring for proton range verification of newly developed iron oxide nanoparticles doped with Zn, we investigated the PET and prompt gamma radiation produced when the samples were irradiated by 10 MeV protons at the CMAM facility [38]. While the PET signal was weak and could only be observed above the background for high concentrations and long periods after irradiation, prompt signals were observed down to lower concentrations of Zn. For prompt measurements, an experimental setup based on four LaBr_3_(Ce) fast scintillator detectors, similar to those described by Vedia et al. [39], and a fully digital data acquisition system that ensures high rate and coincidence capabilities were used. The total full-energy peak efficiency for the detection setup was 1.3(2)% at 662 keV and 0.43(11)% at 1332 keV.

**Figure 4:**
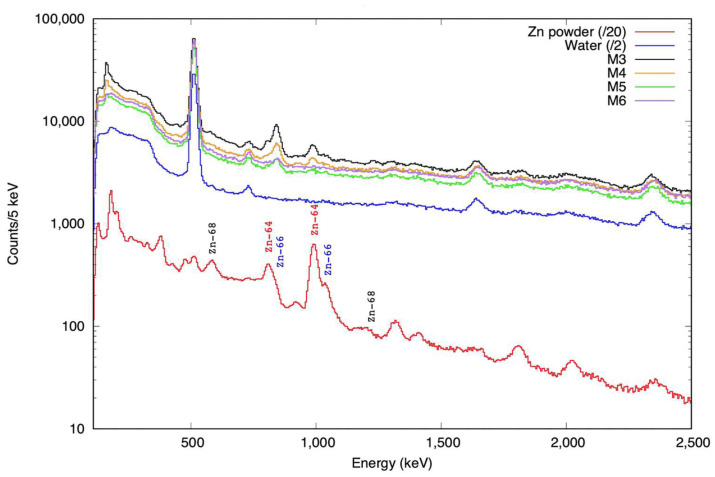
Prompt gamma energy spectra were obtained during the irradiation of the Zn nanoparticle samples with 10 MeV protons for one of the four gamma detectors. Zinc powder (red) was used as a reference. The main prompt gamma rays are labeled, arising from (p,p′γ) reactions on natural Zn isotopes. Water irradiation was used as the background reference (blue). Samples with decreasing concentrations of Zn nanoparticles were labeled as M3 to M6: 80, 40, 20, and 10 mg/mL of Zn, respectively, corresponding to 2.34, 1.17, 0.58, and 0.29 mg of Zn.

The gamma spectra obtained from the irradiation of several samples are shown in Figure 4. A sample of Zn powder (spectrum scaled down by a factor of 20 in Figure 4 for clarity) served as a reference for the identification of the main prompt γ-rays. The irradiated water samples provided information on background components.

**Figure 5:**
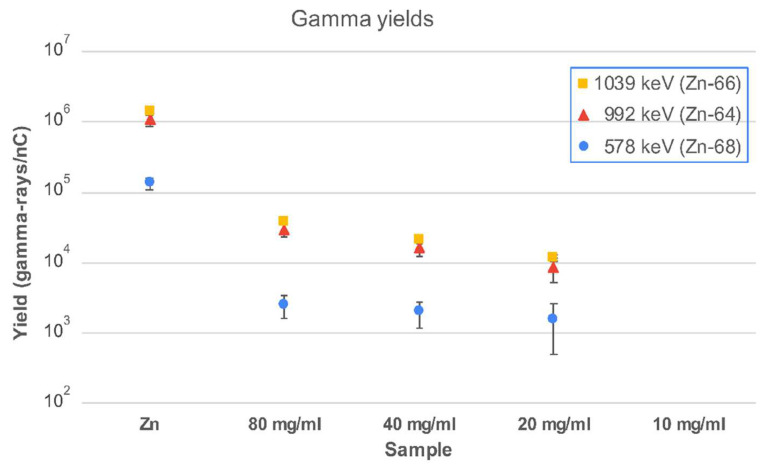
Prompt gamma production yields for selected γ-rays from Zn nanoparticle samples with different concentrations irradiated with 10 MeV protons. See text for details.

The main γ-ray production channels arise from (p,p′γ) reactions on the natural isotopes of Zn, namely ^64^Zn, ^66^Zn, ^67^Zn, and ^68^Zn, with natural abundances of 49%, 28%, 4%, and 19%, respectively [40]. For the analysis, we used the most intense γ-rays at 578, 992, and 1039 keV from the (p,p′γ) reaction on ^68^Zn, ^64^Zn, and ^66^Zn, respectively. The gamma yields, expressed as the number of γ-rays per nC of the integrated proton charge, obtained as the average of the four detectors, are plotted in Figure 5. The expected prompt gamma-ray peaks from IONP@Zn-cit NPs with a small amount of Zn were only observed with a very weak intensity.

### 2.5. Biodistribution and Pharmacokinetic Studies of ^67^Ga-IONP@Zn-cit

**Figure 6:**
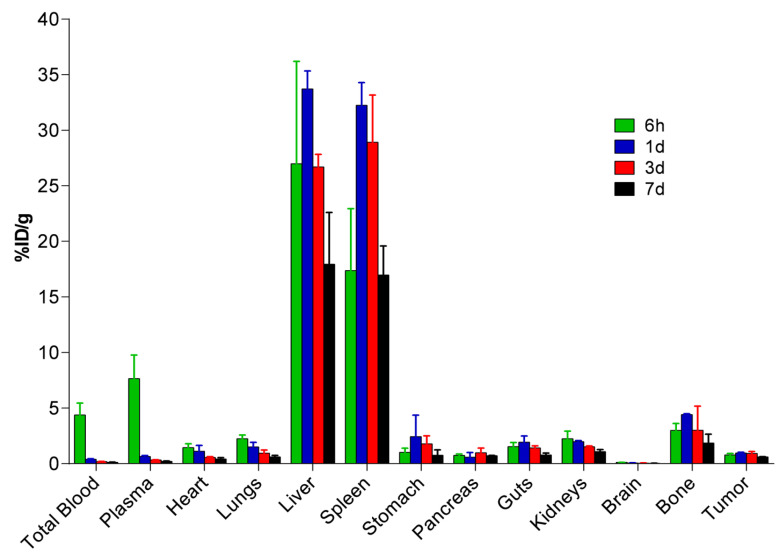
^67^Ga-IONP@Zn-cit biodistribution in a U251 xenograft subcutaneous murine tumor model.

The distribution profile and pharmacokinetics of nanoparticles doped with Zn and labeled with ^67^Ga (^67^Ga-IONP@Zn-cit) were evaluated ex vivo in a U251 xenograft subcutaneous tumor murine model. Mice were euthanized 6 h and 1, 3, and 7 days after intravenous administration of the probe. Different organs and tissues, including the tumor tissue, were measured using a gamma counter, and the results are presented in Figure 6 (detailed values in Table S1). Radioactivity was maintained at similar levels in the tumor tissue over time (0.61–0.95%ID/g). Major ^67^Ga-IONP@Zn-cit accumulation was observed in the liver and spleen, with maximum values at 1 d post-administration (33.72 and 32.24%ID/g, respectively). Moreover, accumulation in the bone was observed, with values ranging between 4.41%ID/g at 1 d and 1.85%ID/g on the last day of the study. Taking the dose and concentration of ^67^Ga-IONP@Zn-cit administrated into account, a maximum value of 1.51 µg Zn/g was achieved after 1 d in the tumors (Table S2). Non-compartmental pharmacokinetic analysis of the plasma data determined that the terminal phase half-life and mean residence time were 3.9 and 2.8 days, respectively. The blood clearance was very low (0.43 mL/d/kg). 

## 3. Discussion

Proton therapy is an emerging modality for high-precision radiotherapy that potentially offers a better dose conformation to the tumor than conventional radiotherapy (photons and electrons), delivering a maximum dose in a well-defined area (Bragg peak) and a rapid dose fall-off beyond that maximum. This allows higher doses of radiation to be delivered to the tumor while sparing the surrounding healthy tissues. To fully utilize the dosimetric advantages of proton beams, it would be highly beneficial to determine the range in which protons reach the tissues of the patient. Therefore, various in vivo techniques have been proposed to verify whether the dose deposited in each treatment session is as expected. However, these techniques have not reached a sufficient level of accuracy to allow their translation to the clinic. To do this, we explored the possibility of using nanoparticle probes with Zn as activatable agents to improve PET and PG signals after proton irradiation as a tool to verify proton range deposition because the presence of metals in nanoparticles enhances the specific gamma signal and improves the detection statistic. Herein, we synthesized a nanoparticle that can be modified for different purposes (e.g., molecular imaging) and evaluated whether these zinc-doped iron oxide nanoparticles capped with citrate (IONP@Zn-cit) could be used as PET and PG signal enhancers. In addition, an important part of this study was to understand how NPs biologically affect tumors before and after irradiation. To achieve this, we performed detailed in vitro and in vivo studies before further investigation. 

First, the effect of proteins present in the medium on NP colloidal stability was evaluated by incubating different concentrations of NPs in the cell culture medium with and without fetal bovine serum (FBS) supplementation. The nanoparticle surface composition determines the initial formation of a protein corona and its behavior [41,42]. Citrate-coated materials have negative surfaces and, hence, potentially more reactive surfaces and antioxidant properties and confer stable synthesis and dispersion [43,44,45,46,47]. Moreover, it has been reported that media composition and ionic strength influence these properties [43]. For example, the use of 10% FBS supplementation or Ca^2+^ and Mg^2+^ present in the cell medium can cause precipitation through preferential complexation with citrate ligands in different types of nanoparticles [44,48,49]. In contrast, other types aggregate in phosphate buffer solution (PBS) but are stable when PBS is supplemented with FBS because of the formation of a corona that stabilizes the MNPs [50]. Albumin proteins of FBS can protect the NP surface from contact with ions [51], and we hypothesize that this last situation could be the case for IONP@Zn-cit in the supplemented culture medium, where they were more stable. When nanomaterials enlarge their size, form aggregates, and/or interact with serum proteins, cellular uptake and toxicity are strongly influenced. Consequently, we studied the cytotoxicity induced by IONP@Zn-cit in the U251 Glioblastoma Multiforme (GBM) cell line. GBM is one of the most common malignant tumors of the central nervous system in adults [52] with the worst prognosis and is a good candidate for treatment with protons to increase the dose in the target volume while protecting organs at risk (OAR) and maintaining cognitive functions [53]. Regarding the cytotoxic effect of nanoparticles, they can be dissolved, producing ion release in the cell culture medium [23,24,46,54,55], or they can act as Trojan horses to produce intracellular release of these ions after cellular uptake, inducing cytotoxicity by oxidative stress and inflammatory signals [43,46,54,56]. The cytotoxicity of metal oxide nanoparticles is generally evaluated based on cell viability, cell membrane damage, oxidative stress, and DNA damage. In many cases, cell viability is measured in terms of mitochondrial enzyme activity using the MTT, XTT, MTS, or WST-1 assays [54]. Other studies have used techniques such as flow cytometry [57]. Our results by MTT assay showed a dose-dependent cytotoxicity effect after 24 h exposure, with an IC50 of 63.80 µg Fe/mL and 99.79 µg Zn/mL obtained, in accordance with the values obtained by Uzar et al. with the MTT assay in NRK-52E cells after 24 h exposure (IC50 of 73.05 µg/mL for ZnO) [58]. Thurber et al. studied the effect of the Fe doping levels of ZnO nanoparticles on cell viability by the Alamar Blue cytotoxicity assay. They obtained significantly greater toxicity for low Fe doping levels (<10% Fe) compared to undoped ZnO NPs (IC50 values of 228 µg/mL and 370 µg/mL, respectively). This indicates the strong role of Fe ions in increasing the cytotoxicity of the ZnO NP. Interestingly, pure iron oxide (Fe_2_O_3_) showed negligible cytotoxicity [59]. In our study, the results were similar for metal doping with Zn. ^68^Ga-C-IONP, which was our equivalent nanoparticle, but without Zn, was tested using image-based high-content analysis, with no reduction in the number of cells in different cell lines [34]. Ferroptosis has been defined as a new type of cell death in which Fe(II) ions from NPs react with hydrogen peroxide and produce cytotoxic free reactive oxygen species (ROS) through the Fenton reaction [50,60,61], similar to the presence of Zn ions from NPs [24,59]. Wang et al. observed how ZnO NPs repressed expression of ferroptosis-negative regulatory factors, reducing the cell viability of several cell lines by colony formation assays [62] and pointing out the possible roles of zinc in ferroptosis, also studied in other publications [63,64]. In conclusion, based on the definition of Horie et al. (who defined that a nanoparticle was cytotoxic when the LD50 was <100 µg/m [54]), we could describe IONP@Zn-cit as a cytotoxic nanoparticle, but further investigations are necessary to determine how it could affect the use of this nanoparticle as a proton activatable agent and what their cytotoxicity mechanism is. Several possibilities have been proposed, such as quantifying Fe and Zn ions in the medium and in the cells and their cellular uptake by different techniques, such as ICP [65], colorimetric assays [47,66], TEM microscopy, and radioactivity detection [67]. 

Furthermore, we tested the effect of non-toxic IONP@Zn-cit concentrations on the clonogenic survival of tumor cells in the presence of X-ray irradiation. Metallic nanoparticles can be used as traceable agents in medical imaging modalities or to enhance radiation treatment at lower costs [22,28,67,68]. The radiosensitization produced by nanoparticles depends on numerous factors, including the cell line, nanoparticle type and size, concentration, intracellular localization [69], and the energy and nature of incoming radiation. However, the underlying mechanisms leading to increased cell death remain unclear [28]. Physical, chemical, and biological mechanisms have been proposed, including the generation of high-density reactive oxygen species (ROS), cell cycle effects, and DNA repair impairment [29,69,70]. According to the data obtained from the clonogenic survival assay without X-irradiation, a decrease in the number of colonies and cell surviving fraction was observed in the presence of IONPs@Zn-cit, although non-toxic concentrations were used (according to the MTT assay). It is important to highlight that metabolic state and clonogenic potential are not necessarily parallel events, such that prematurely senescent cells do not form colonies but are metabolically active. Deylam et al. described that zinc oxide nanoparticles increase the senescence of cells treated with NPs containing 6–12 µg Zn/mL [71]. Moreover, we found that treatment with 1 Gy X-ray irradiation failed to decrease the percentage of living cells in the cell populations that were exposed to Zn-NPs compared to cells without nanoparticles. This is a strong indication of the acquisition of radioresistant properties. One possible explanation for this finding might be that non-proliferative senescent cells are less sensitive to irradiation, as it is known that non-proliferating cells are less susceptible to irradiation due to their altered metabolism.

A decrease in the α value and an increase in the β value were observed in U251 cells incubated with IONP@Zn-cit nanoparticles after irradiation. Due to the rarely used α and β values to evaluate nanoparticle effects, researchers have proposed several theoretical tools to measure the nanoparticle-mediated effect: Endpoints for clonogenic survival such as SF_2Gy_, SF_4Gy_, SF_6Gy_, SF_8Gy_, D_10%_, and D_50%_ [72], dose modifying factor or DMF [73], sensitizer enhancement ratio or SER [74,75], dose enhancement ratio or DER [67,76,77,78], mean inactivation dose or MID [65,79], dose modifying ratio or DMR_x%_ and dose enhancement factor or DEF_xGy_ [28,65], among others. We decided to use SF_2Gy_, D_50%_, and D_10%_ as endpoints; DEF_2Gy_ to evaluate the effect at 2 Gy (a commonly used clinical dose); and MID, which is representative of the whole cell population, minimizes the fluctuations in the survival curves and takes into account the whole survival curve [65]. An increase in the values of all these indicators was observed in cells incubated in the presence of the NPs, suggesting a radioprotective effect of IONP@Zn-cit, which has already been described for low iron and zinc doses owing to their chelation properties and metabolism [80,81] and the effect of Zn in reducing ferroptosis [82]. Moreover, some nanoparticle coatings, such as citric acid (coating agent in IONP@Zn-cit), are well-known hydroxyl radical scavengers that can contribute to maintaining an optimum physiological level of ROS in cells [65]. Finally, it is important to emphasize that these clonogenic survival assays with X-ray irradiation have been a proof of concept to progress, and further investigations should be conducted with different irradiation modalities and energies (protons and clinical photons) because of the possible differences in the results [73,83]. 

Different concentrations of IONP@Zn-cit were irradiated with 10 MeV protons to evaluate the production of PET and prompt gamma signals. Although the PET signal was negligible, detectable prompt gamma activity could be observed down to 10 mg Zn/mL with our reduced efficiency detector setup, holding the potential to improve it for the detection of lower concentrations. Furthermore, we evaluated the biodistribution of the NP in a subcutaneous U251 mouse model. Our ^67^Ga-IONP@Zn-cit accumulation profile throughout the body was also consistent with typical nanoparticle biodistribution, in which reticuloendothelial (RES) organs are the major clearance route for nanoparticles larger than 5 nm. RES is part of the immune system of the body, being a network of cells and tissues, especially in the blood, general connective tissue, spleen, liver, lungs, bone marrow, and lymph nodes [26,27,37]. The mononuclear phagocytic system (MPS) is responsible for its accumulation in the liver and spleen. Owing to the small IONP@Zn-cit size (10 nm) determined by characterization studies, more rapid elimination would be expected through the kidneys [27]. However, our results of colloidal stability in supplemented medium and the biodistribution profile suggest the formation of a larger protein corona in the bloodstream. The relatively high accumulation in the bone and stomach observed compared with other tissues could be explained by ^67^Ga accumulation because of ^67^Ga-IONP@Zn-cit metabolism [84,85,86]. Moreover, we precisely determined a terminal phase half-life of 3.9 days thanks to the ^67^Ga half-life (T1/2: 78.3 h), which allows a biodistribution and pharmacokinetic study up to 7 days post-injection. These results indicate a slow clearance of IONP@Zn-cit compared to other iron oxide nanoparticles, with t1/2 values of approximately minutes or hours [27,37]. This longer blood circulation time would allow increased tumor accumulation and could avoid washout problems that occur with other contrast agents or molecules.

Nevertheless, cytotoxicity should be studied because of long-term tissue exposure to these nanoparticles [36,37]. The obtained values for tumors were consistent with passive delivery and accumulation due to the enhanced permeability and retention (EPR) effect [27,87] and were comparable to those published by Pellico et al. with ^68^Ga-C-IONP nanoparticles [34], which were approximately 2% ID/g. In our case, 0.95 ± 0.08% ID/g supposes 1.5 µg Zn/g tissue at 1-day NPs post-injection. These results show the need for higher Zn concentrations in tumor tissues than those obtained with our NPs to improve PET and PG signal sensitivities and to evaluate the range of PRT. To achieve this, several strategies can be adopted. The first proposal is to increase the amount of NP administrated. We injected 4.8 mg Zn/kg and 3.2 mg Fe/kg of ^67^Ga-IONP@Zn-cit for the in vivo study. Other researchers used a maximum NP injection of 300–600 mg Fe/kg body when toxicity appeared [37]. Moreover, the amount of Zn due to passive targeting by the EPR effect could be improved by changing some parameters, such as the shape of the nanoparticles, stealth capacity of the nanoparticles, and surface charge. For example, the presence of PEG molecules at the nanoparticle surface leads to a prolonged circulation half-life, reduced protein adsorption, and a reduced clearance by MPS, thus improving tumor accumulation [27]. However, passive targeting has the following limitations: The inhomogeneous distribution of blood vessels resulting from angiogenesis, which yields non-uniform permeability within the whole tumor [27,70], and its lack of tumor specificity [27]. Active targeting could be an option in the future to improve our results with the use of molecules such as antibodies or peptides specific to the tumor tissue [34], which could increase IONP@Zn-cit accumulation. When IONP@Zn-cit was used as a proton verification range for glioblastoma multiforme disease, our nanoparticles did not pass across the blood–brain barrier (BBB), as can be seen in the results obtained with the biodistribution study. Several mechanisms have been proposed to modify IONP@Zn-cit to overcome this barrier, including conjugation with a simil-opioid glycopeptide (g7) [88], among others [89]. Finally, another strategy to enhance the IONP@Zn-cit PG signal, in addition to increasing the sensitivity of prompt gamma detection by including a higher number of LaBr_3_(Ce) detectors [20], could be the addition of oxygen-10-enriched water during NP synthesis instead of conventional water to obtain several isotopes with high proton-induced reaction cross-sections in the same contrast agent for proton range verification [15]. 

In summary, we presented a new iron oxide nanoparticle doped with Zn (IONP@Zn-cit) obtained using an easy, quick, and reproducible synthesis method for proton range verification. IONP@Zn-cit can be easily modified in several ways to obtain a contrast agent suitable for diagnostic imaging (MRI, PET, and SPECT) and treatment. In the present study, not only physical and chemical studies were carried out to characterize the nanoparticles but also biological tests that allowed the study of their biodistribution and toxicity, which aimed to determine how nanoparticles could be transferred to the clinic. The obtained results are encouraging; however, future preclinical studies using nanomaterials for proton range verification are needed to fully understand and exploit the benefits associated with their use. In addition to the characterization of nanoparticles, it is essential to carry out studies on their stability, cytotoxicity, effect in combination with different radiation modalities, biodistribution, and pharmacokinetics.

## 4. Materials and Methods

### 4.1. Nanoparticle Synthesis

#### 4.1.1. IONP@Zn-cit

**Figure 7:**
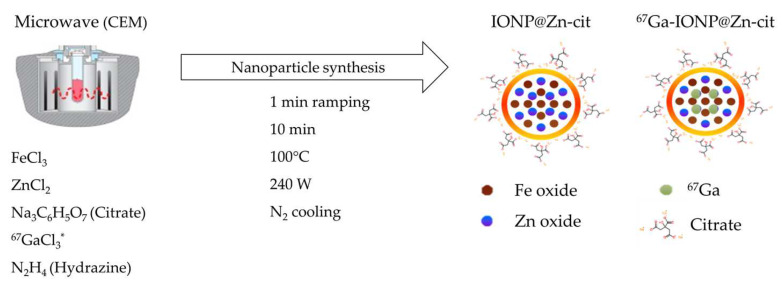
Different types of nanoparticles used in this study and their synthesis. As initial products, FeCl_3_, ZnCl_2_, citrate and hydrazine were used and microwave irradiation during 10 min at 100 °C with a final cooling step was done to synthesize IONP@Zn-cit. * In the case of ^67^Ga-IONP@Zn-cit, ^67^GaCl_3_ was added to the initial mixture.

IONP@Zn-cit was synthesized by combining 25 mg FeCl_3_, 25 mg ZnCl_2_, 58.5 mg citrate trisodium (selected as a coating to ensure colloidal stability), and 0.5 mL hydrazine hydrate in H_2_O milliQ at a final volume of 5 mL. Immediately, the mixture was subjected to very fast ramping (1 min) to 100 °C under stirring with microwave irradiation at 240 W (MW; CEM) for 10 min, and a final cooling step with N_2_ gas until 55 °C was achieved (Figure 7). Subsequently, 6 mL of IONP@Zn-cit was purified by gel filtration using PD-10 desalting columns (Sephadex G-25 Medium; Cytvia) in water. The concentrations of incorporated Fe and Zn were determined by ICP-MS. For the in vitro studies (colloidal stability, cytotoxicity, and clonogenic assays), nanoparticle suspension medium was exchanged by ultrafiltration (Amicon Ultra-15, membrane PLTK Ultracel-PL, 30 kDa; Merck Millipore) at 1520× *g* for 3 min. After that, the cell culture medium was added to resuspend the nanoparticles, and the centrifugation step was repeated twice until the medium was adjusted to the initial volume to maintain concentrations. Finally, the NPs were sterilized using a vented Millex-GS filter of 0.22 µm (Merck Millipore).

#### 4.1.2. Radiolabeled ^67^Ga-IONP@Zn-cit

For the Gallium-67 radiolabeled probe used in the in vivo study, 36.48 MBq of ^67^Ga-citrate (T1/2: 78.3 h; γ-rays: 93 keV (40%), 184 keV (24%), 296 keV (22%), and 388 keV (7%) [90]) was purchased from Curium Pharma Spain S.A. ^67^Ga-citrate was transformed to ^67^GaCl_3_ by an ionic exchange step to achieve good incorporation of ^67^Ga in the IONP@Zn-cit structure. Silica Gel MiniSpe-ed™ Cartridges (14014; Applied Separations) and HCl 0.1 M as eluent were used; 0.2 mL fractions were collected, and 0.8 mL in total were selected as peak activity (fractions 4th to 7th). H_2_O milliQ and HCl 12.07 M (Hydrochloric acid fuming 37%; Merck) were added to adjust molarity and obtain 1 mL of ^67^GaCl_3_ in 1 M HCl. This was added to the mixture of 25 mg FeCl_3_, 25 mg ZnCl_2_, 58.5 mg citrate trisodium, and 3.5 mL of H_2_O milliQ. Finally, 0.5 mL hydrazine hydrate was added immediately before the mixture was subjected to MW irradiation under the same conditions described above (Figure 7). Purification was performed by size exclusion chromatography with PD-10 desalting columns in 0.5 mL fractions to obtain 6 mL of purified ^67^Ga-IONP@Zn-cit in water. The activity of the eluted fractions was measured using an activimeter (VCC-405; Veenstra Instruments) to obtain the peak activity between fractions 2 and 7. For in vivo studies, NPs were ultrafiltered with Amicon filters at 1520× *g* for 3 min, and saline (0.9% NaCl) was added between each centrifugation step to suspend nanoparticles and to adjust the initial volume to maintain initial concentrations at the end. Finally, sterilization was performed using a vented Millex-GS filter of 0.22 µm (Merck Millipore). The physicochemical properties of the different types of nanoparticles, such as surface charge and size, DLS measurements, TEM images, and ICP-MS quantification of Fe and Zn, were characterized after ^67^Ga decay. 

### 4.2. Nanoparticle Characterization

#### 4.2.1. Hydrodynamic Size and Zeta Potential Measurements

The hydrodynamic size and Zeta potential of the samples were measured by dynamic light scattering (DLS). A Zetasizer Nano ZS (Malvern Instruments, Malvern, UK) instrument was used. This device is equipped with a He−Ne laser operating at 633 nm and 4 mW, and an avalanche photodiode detector.

#### 4.2.2. TEM Images

Sample preparation was carried out by dripping a nanoparticle suspension onto a carbon-coated copper TEM mesh grid, allowing for solvent evaporation. In this case, a JEOL JEM 2100 with a 3 Å resolution operating at 200 kV at the Spanish National Electronic Microscopy Center (CNME) was used.

### 4.3. In Vitro Studies

#### 4.3.1. IONP@Zn-cit Colloidal Stability in Cell Culture Medium

Different concentrations of IONP@Zn-cit (between 20.81 ± 0.84 and 665.75 ± 26.87 µg Zn/mL) were incubated in DMEM (1×) + GlutaMax (Gibco, Billings, MT, USA) cell culture medium with and without 10% of fetal bovine serum (FBS) at 37 °C for 3 h or 7 days. Aggregation and instability were evaluated visually.

#### 4.3.2. Cell Culture

The U251 cell line established from a human malignant glioblastoma multiforme (provided by Dra. Francisca Mulero, CNIO, Madrid, Spania) was cultured under sterile conditions in DMEM (1×) + GlutaMax (Gibco) supplemented with 10% fetal bovine serum (FBS; Sigma, Burlington, MA, USA) and 1% penicillin/streptomycin (10,000 U penicillin + 10 mg/mL streptomycin; 100 mL; Sigma). The cells were maintained at 37 °C in 5% CO_2_ and 95% humidity conditions until 80–90% confluence was reached before each experiment.

#### 4.3.3. Cytotoxicity Assay

The MTT assay was performed on U251 cells. The cells were seeded at a density of 20,000 cells/well in 96-well culture plates. After 24 h, the medium was changed to fresh medium containing different nanoparticle IONP@Zn-cit concentrations (from 25 to 150 µg Zn/mL) and cells were incubated for another 24 h with the nanoparticles under normal culture conditions. Then, the medium was aspirated, followed by two washes with 1× DPBS (Dulbecco’s Phosphate Buffered Saline; Sigma), and Cell Proliferation Kit I (Roche, Basel, Switzerland) was used to assess the metabolic activity of the cells. Viable cells with active metabolism convert MTT into formazan, whose absorbance at 570 nm is proportional to the number of viable cells [91,92]. The plate was read at 570 nm (formazan absorbance) and 690 nm (background absorbance) using a FLUOStar^®^ Omega microplate reader (BMG LABTECH, Ortenberg, Germany). The concentration of nanoparticles that inhibited 50% of the cells (IC50) was calculated using a sigmoidal curve adjustment. The experiments were repeated three times in triplicate.

#### 4.3.4. Colony Formation Assay

We conducted a clonogenic assay as the gold standard for measuring the effect of nanoparticles on cells after radiation exposure. U251 cells were grown under normal culture conditions named above in a 25 cm^2^ cell culture flask (Thermo Scientific™ Nunc™ EasYFlask™, Waltham, MA, USA). After growth, the medium was replaced with fresh medium containing different concentrations of IONP@Zn-cit (0, 1, and 10 µg Zn/mL) at each X-ray irradiation dose. After 24 h of incubation with NPs under normal cell culture conditions, the flasks were irradiated with the corresponding doses (1.01 ± 0.01, 5.22 ± 0.04, or 10.39 ± 0.07 Gy, simplified in the main text as 1, 5, and 10 Gy). A clonogenic assay was performed by plating cells after irradiation treatment with a delay of 4 h (Delayed Plating or DP) to allow repair processes [93], after which the cells were washed twice with 1× DPBS to remove non-internalized NPs and harvested with TrypLE™ Express (Gibco) to prepare single-cell suspensions. At this time, cells were counted with a hemocytometer and seeded in different cell amounts in triplicate depending on the irradiation dose (100–200, 500–1000, and 2000–4000 cells/well for 0–1, 5, and 10 Gy, respectively) at a final volume of 2 mL per well in 6-well plates (Nunc). Cells were cultured under normal conditions until they reached clusters of ≥ 50 cells and were visible at first sight (10 d after seeding). At this point, the cell culture medium was removed and a fixing/staining solution (0.05% *w*/*v* crystal violet, 1% formaldehyde 37%, 1× PBS, 1% methanol, and dH_2_O) was added to each well until complete coverage (2 mL approximately) and incubated for 20 min at room temperature. Finally, the solution was removed and washed with an indirect tap water rinse. This experiment was performed in triplicate and repeated four times. When plates were completely dry (after approximately 24 h at room temperature), digitalization with an Epson Expression 10,000XL scanner was performed (48-bit images were taken at 1200 ppp resolution, 1.5 focus, in transmission mode, and TIF format). For quantification, ImageJ software v1.52a [94] was used to count colonies. For 8-bit image transformation, Threshold Above was selected manually for each plate to obtain a proper image with defined colonies, and Close-, Erode, and Watersheld tools in this order were selected for processing binary images to avoid over- and under-estimated colony counting. The number of colonies was obtained with the Analyze Particles tool using a minimum particle size of 200 pixel^2^ and a circularity of 0.12–1.00 as parameters, defined by experience. The plating efficiency percentage (PE %) of the non-irradiated group (control) was calculated for each well, and the number of cells seeded was calculated as follows:

(1) $$PE\left(\%\right)=\frac{No.\;colonies\;scored}{No.\;cells\;seeded}\times 100,$$

Survival fraction (SF) after each irradiation treatment was calculated for each well and each amount seeded for each NP treatment with the expression:

(2) $$SF=\frac{\frac{No.\;colonies\;scored}{No.\;cells\;seeded}\times 100}{PE(\%)},$$

All results were obtained automatically with an MS Excel template file created by Brix et al. [95]. Survival curves were generated by plotting the SF versus the radiation dose and fitting to the traditional linear-quadratic (LQ) regression model (Equation (3)) for each experiment and NP concentration (0, 1, and 10 µg/mL). Regression analysis was performed using the following equation:

(3) $$SF\left(D\right)={exp}^{-\alpha D-\beta D^{2}},$$

where SF is the cell survival fraction, α is the probability of lethal DNA damage (linear part of the curve), β is the probability of sublethal damage (quadratic part of the curve), and D is the radiation dose (Gy). Curves were weighted with the inverse of variance using OriginPro, Version 8 SR4 software (OriginLab Corporation, Northampton, MA, USA). The survival fraction after a dose of 2 Gy (SF_2Gy_) and the dose resulting in 10% and 50% survival (D_10%_ and D_50%_) were calculated as biological endpoints with less noise than α and β values [96]. The mean inactivation dose (MID) was used to calculate the differences between the survival curves of the treatment groups. The main advantage of defining MID is that it considers the whole survival curve, which eliminates the source of the fluctuation survival curve. The concept of MID introduced by Kellerer and Hug is defined as the area under the survival curve [65,79]:

(4) $$MID=\int_{0}^{\infty }SF\left(D\right)dD,$$

Finally, the initial slope of the survival curve correlates well with clinical outcomes, and this region is considered to be best characterized by the survival level at a dose of 2 Gy [65]; moreover, 2 Gy is the typical individual dose of conventional radiotherapy fractionation delivery. Therefore, to evaluate the effect of IONP@Zn-cit NPs in a well-defined scenario, the Dose Enhancement Factor (DEF_2Gy_) was determined as the ratio of the dose required for NP-treated cells to give the same survival as cells not treated with NPs and irradiated at 2 Gy, where the ratios were determined using the LQ fits [28,65]:

(5) $${DEF}_{2Gy}=\frac{Reference\;dose\;(2\;Gy)}{\begin{array}{c} Dose\;needed\;with\;\left(cells+NPs\right)to\;achieve\;the\;same\;cell\;survival\\ as\;cells\;alone\;at\;a\;dose\;of\;2\;Gy \end{array}}$$

### 4.4. Irradiations

#### 4.4.1. X-ray Irradiation

For the clonogenic assay, the X-ray irradiator used was a Philips MCN 321 X-ray tube with a Philips MG324 generator. The irradiation conditions were: 250 kV with 10 mA; the inherent filtration was 2.2 mm of Be with 3 mm of Al; with an additional filtration corresponding with a Thoraeus I filter; the half-value layer (HVL) of (2.3 ± 0.3) mm for Cu was measured by an experimental procedure. Absolute dose calibration was performed based on the TRS-398 protocol using a 0.6 cm^3^ Farmer ionization chamber (model NE-2571). It was calibrated at the Metrology Laboratory for Ionizing Radiation of the CIEMAT. The dose for the cell configuration was measured with a Farmer Ionization Chamber in an equivalent phantom; however, an EBT-3 radiochromic film was calibrated and used to check the dose at the cell plane using the real geometry. The dose rate obtained was 0.58 Gy/min, with a 4% uncertainty. To dismiss the uncertainty between measurements due to the instability of the X-ray and the time imprecision in each irradiation, we used a 0.6 cm^3^ Farmer Ionization Chamber as the monitoring chamber at a point inside the beam for each irradiation, and the assigned dose to the cells was corrected based on that measurement (Figure S3).

#### 4.4.2. Proton Irradiation

Irradiation to investigate PET and prompt gamma radiation produced by IONP@Zn-cit were conducted at the external microbeam beamline at CMAM with a proton energy of 10 MeV and a current intensity of 500 pA. Prompt gamma radiation was detected using a simplified detection setup consisting of four LaBr_3_(Ce)-based detectors read out by a digital data acquisition system (Figure S4), similar to those described by Vedia et al. [39]. The combined full-energy peak efficiencies for the four detectors were 1.3(2)% at 662 keV and 0.43(11)% at 1332. keV. An offline station with two more detectors of the same type was added for the decay measurements of PET isotopes produced by activation. 

Beam alignment was performed with an Eppendorf tube at 3 MeV at both the entrance and exit of the tube, using a radiochromic film. Nanoparticles were used in the aqueous solutions discussed above with varying concentrations. A sample of Zn powder served as a reference for the identification of the main prompt γ-rays, and the irradiated water sample provided information on the background components.

### 4.5. In Vivo Studies

#### 4.5.1. Animal Model

All animal experimental protocols were approved by the local Animal Care and Ethics Committee and regional authorities. For the subcutaneous tumor xenograft model, 1 × 10^6^ U251 cells were suspended in 0.1 mL of a mixture of non-supplemented DMEM (1×) + GlutaMax medium with cold Matrigel (Corning^®^ Basement Membrane Matrix; Cultex) at a 1:1 ratio [97]. They were then implanted with a sterile cold 25 G syringe into the right flank of male nude mice (*n* = 8; 16-week-old). Animals were maintained at 22 °C in a 12 h light/dark cycle with water and food available ad libitum. Tumors were palpable approximately 2 weeks post-implantation.

#### 4.5.2. Biodistribution Study

The biodistribution of ^67^Ga-IONP@Zn-cit was evaluated in the animal model described. A nanoparticle volume of 0.15 mL (0.62 ± 0.02 MBq; 158 µg Zn and 103.5 µg Fe) in saline (NaCl 0.9%; B. Braun) was injected intravenously into the tail vein under anesthesia (2–3% isofluorane, 0.6–0.8 L/min O_2_). Then, the mice were weighed and euthanized by cervical dislocation at each time point (6 h, 1, 3, and 7 days post-injection). Blood samples were immediately collected by cardiac puncture using a heparinized syringe, weighed, and centrifuged at 3000× *g* for 8 min to obtain plasma and the rest of the blood. Mice were dissected, and their organs and tissues (heart, lungs, liver, spleen, stomach, pancreas, gut, kidneys, brain, right femur, and tumor) were extracted, weighed, and measured using a gamma counter (2470 Wizard^2^, PerkinElmer, Waltham, MA, USA). 

Plasma concentrations of radioactivity were calculated as the percent injected dose per mL (%ID/mL) and plotted versus time post-injection. Non-compartmental pharmacokinetic analysis of plasma data was performed using PK Solver software, version 2.0 [98].

### 4.6. Statistical Analysis

OriginPro, Version 8 SR4 software (OriginLab Corporation, Northampton, MA, USA) was used for data analysis. All data are expressed as the mean ± standard deviation. The Shapiro–Wilk test was used to assess data normality. One-way multivariate analysis of variance (ANOVA) was performed using SPSS 14; Tukey or Games–Howell post hoc tests were used to compare the differences between groups when equal variances were assumed (assessed by Levene’s test of homogeneity of variances) or not. *p* < 0.05 was considered to be statistically significant.

## 5. Conclusions

The aim of this study was to develop an innovative methodology based on Zn nanoparticles to address the shortcomings of in vivo monitoring of the range of protons in proton therapy. To achieve this, IONP@Zn-cit was synthesized, and its physicochemical properties and biological characteristics were studied.

The results showed that Zn-doped iron oxide nanoparticles capped with citrate were biocompatible and stable in the presence of serum. Their cytotoxicity allows for in vitro studies, where we observed a possible radioprotective effect at low doses of nanoparticles under X-ray irradiation. Moreover, the determination of passive accumulation in tumor tissue and other organs in an animal model using a radiolabeled form of the nanoparticle showed the potential of these probes and the importance of further investigation of their biological properties. The observation of the prompt gamma signal after 10 MeV proton irradiation also encourages the study of the potential of nanoparticles in the field of proton range verification.

## Figures and Tables

**Table 1:** IC50 values are in µg/mL for each metal (Fe and Zn) and both metals together, with their 95% confidence intervals.

NP Component	IC50 (μg/mL)	95% Confidence Intervals
Fe	64	61 to 67
Zn	100	96 to 104
Fe + Zn	164	157 to 171

**Table 2:** Radiobiological endpoints of U251 cell survival curves fitted to the LQM. α and β values are expressed as mean ± standard deviation.

[IONP@Zn-cit] (µg Zn/mL)	α (Gy^−1^)	β (Gy^−2^)	SF_2Gy_	D_50%_ (Gy)	D_10%_ (Gy)	MID
0	0.527 ± 0.286	−0.005 ± 0.028	0.355	1.33	4.55	2.62
1	0.206 ± 0.162	0.019 ± 0.016	0.613	2.69	6.82	3.11
10	0.056 ± 0.010	0.039 ± 0.001	0.766	3.57	7.03	2.91

## Data Availability

The data presented in this study are available on request from the corresponding authors.

## Supplementary Materials

The following are available online at https://www.mdpi.com/article/10.3390/molecules28196874/s1. Figure S1. Visual follow-up of zinc-doped iron oxide nanoparticles capped with citrate (IONP@Zn-cit) in concentrations between (20.81 ± 0.84 and 665.75 ± 26.87 µg Zn/mL) after 3 h and 7 days incubations in cell culture medium with and without FBS supplementation. Arrows point phase changes produced by nanoparticle precipitation. Figure S2. Radio-chromatogram of ^67^Ga-IONP@Zn-cit purification after microwave synthesis. Activity (in MBq) present in the fractions (1–15) obtained after purification of the ^67^Ga-IONP@Zn-cit synthesis mixture by gel filtration with PD-10 columns (Se-phadex G-25M). The first peak corresponds to purified ^67^Ga-IONP@Zn-cit and the second one to free gallium-67 in chloride form. Table S1. ^67^Ga-IONP@Zn-cit biodistribution values of different tissues after each time point (6 h, 1, 3, and 7 days), calculated as percentage of injected dose per gram of tissue (%ID/g) with the standard deviation (SD) between animals. Table S2. Accumulation of radioactivity (in %ID/g, expressed as mean ± SD) and calculated ^67^Ga- IONP@Zn-cit (in µg Zn or Fe per gram) in tumor tissues after times (0.25, 1, 3, and 7 days) post probe administration (^67^Ga-IONP@Zn-cit containing 158 µg Zn and 103.5 µ Fe). Figure S3. (a) General view of the irradiation system with the X-ray tube (1). An ionizing chamber (2) was used for dose irradiation control measurements. (b) Top view of the position of four 25 cm^2^ Flask bottles (3) to perform clonogenic assays with nanoparticles. The bottles were forming a square at the same distance from the center of the beam to have the same irradiation dose for all cells seeded. Figure S4. (a) Scheme of the general view of the irradiation system with the proton beam and the four LaBr_3_(Ce)-based detectors. (b) Top view of the real position of a sample in front of the beam and between the detectors.
