# Supplementary material for: Zinc-Doped Iron Oxide Nanoparticles as a Proton-Activatable Agent for Dose Range Verification in Proton Therapy
Source: Molecules. 2023 Sep 29;28(19):6874. doi: 10.3390/molecules28196874 (PMC10574368; doi:10.3390/molecules28196874)
Supplement: Supplementary file 1 [file molecules-28-06874-s001.zip › molecules-2540385-supplementary.pdf]

molecules

MDPI

Supplementary Materials

Zinc-doped iron oxide nanoparticles as a proton-activatable agent for dose range verification in proton therapy

1. IONP@Zn-cit colloidal stability in cell culture medium

IONP@Zn-cit (μgZn/mL)	20.81	41.61	83.22	166.44	332.88	665.75
SD	0.84	1.68	3.36	6.72	13.44	26.87

Incubation time	Medium
3 hours	No FBS
	10% FBS
7 days	No FBS
	10% FBS

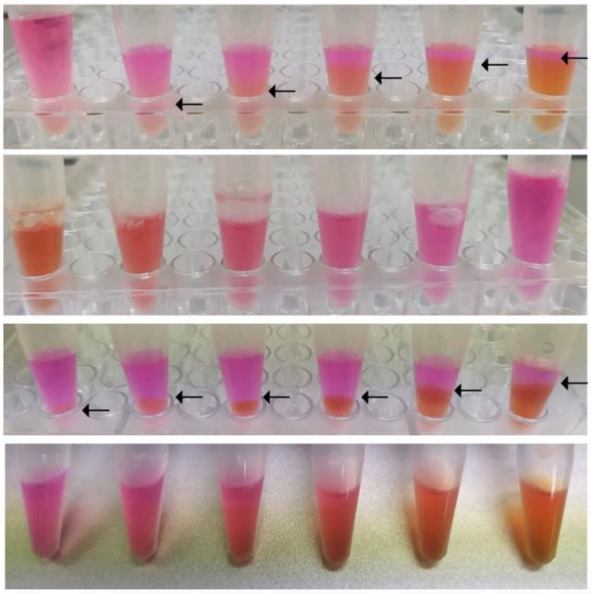

Figure S1. Visual follow-up of zinc doped iron oxide nanoparticles capped with citrate (IONP@Zn-cit) in concentrations between (20.81 ± 0.84 and 665.75 ± 26.87 μg Zn/ml) after 3 hours and 7 days incubations in cell culture medium with and without FBS supplementation. Arrows point phase changes produced by nanoparticle precipitation.

2. Radioactivity profile of ^{67}Ga after ^{67}Ga-IONP@Zn-cit purification

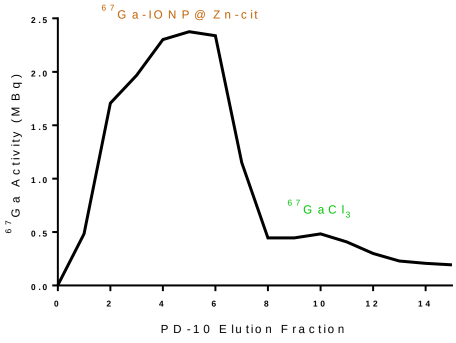

Figure S2. Radio-chromatogram of ^{67}Ga-IONP@Zn-cit purification after microwave synthesis. Activity (in MBq) present in the fractions (1-15) obtained after purification of the ^{67}Ga-IONP@Zn-cit synthesis mixture by gel filtration with PD-10 columns (Sephadex G-25M). The first peak corresponds to purified ^{67}Ga-IONP@Zn-cit and the second one to free gallium-67 in chloride form.

3. Biodistribution and pharmacokinetic studies of ^{67}Ga-IONP@Zn-cit

Table S1. ^{67}Ga-IONP@Zn-cit biodistribution values of different tissues after each time point (6 hours, 1, 3, and 7 days), calculated as percentage of injected dose per gram of tissue (%ID/g) with the standard deviation (SD) between animals.

**Time**	**6h**		**1d**		**3d**		**7d**	
Tissues	*%ID/g*	*SD*	*%ID/g*	*SD*	*%ID/g*	*SD*	*%ID/g*	*SD*
**Total Blood**	4.382	1.061	0.389	0.051	0.187	0.024	0.126	0.025
**Plasma**	7.642	2.123	0.646	0.087	0.320	0.036	0.208	0.037
**Heart**	1.441	0.352	1.118	0.520	0.558	0.069	0.410	0.137
**Lungs**	2.244	0.331	1.505	0.408	0.945	0.288	0.605	0.143
**Liver**	26.983	9.220	33.718	1.616	26.692	1.133	17.934	4.664
**Spleen**	17.346	5.581	32.238	2.050	28.915	4.266	16.958	2.616
**Stomach**	1.016	0.377	2.426	1.942	1.786	0.711	0.759	0.494
**Pancreas**	0.747	0.121	0.588	0.421	0.978	0.425	0.728	0.020
**Guts**	1.528	0.372	1.925	0.557	1.410	0.194	0.781	0.167
**Kidneys**	2.249	0.673	1.977	0.103	1.511	0.076	1.076	0.195
**Brain**	0.116	0.010	0.062	0.026	0.050	0.023	0.036	0.009
**Bone**	2.989	0.620	4.412	0.072	2.997	2.171	1.849	0.803
**Tumor**	0.793	0.126	0.951	0.080	0.921	0.168	0.613	0.024

Table S2. Accumulation of radioactivity (in %ID/g, expressed as mean±SD) and calculated ^{67}Ga- IONP@Zn-cit (in μg Zn or Fe per gram) in tumor tissues after times (0.25, 1, 3 and 7 days) post probe administration (^{67}Ga-IONP@Zn-cit containing 158 μg Zn and 103.5 μ Fe).

	**Tumor Accumulation**	**^{67}Ga- IONP@Zn-cit Concentrarion**	
**Time (days)**	*%ID/g*	*μg Zn/g*	*μg Fe/g*
**0.25**	0.79±0.13	1.26	0.82
**1**	0.95±0.08	1.51	0.98
**3**	0.92±0.17	1.46	0.95
**7**	0.61±0.02	0.97	0.63

4. X-ray Irradiation

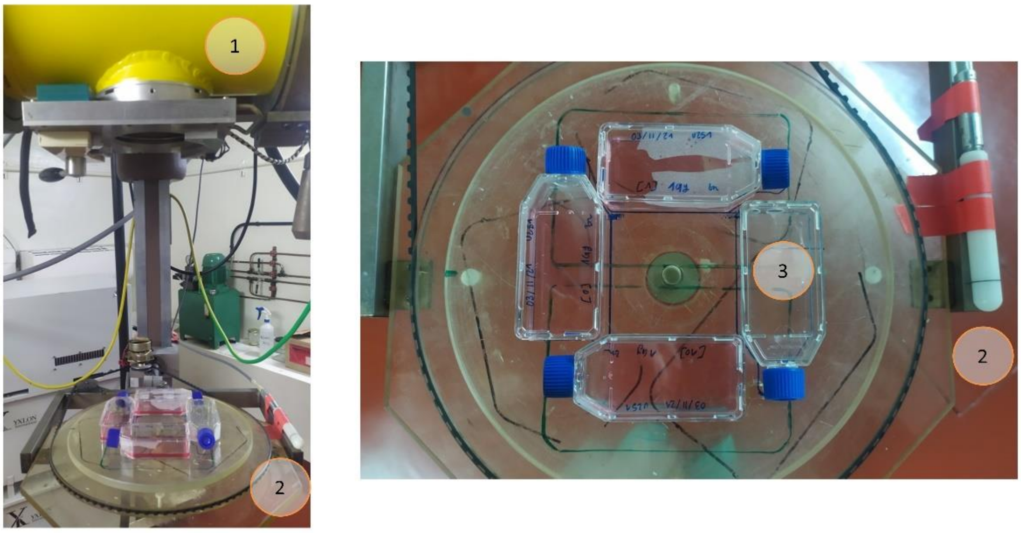

(a) (b)

Figure S3. (a) General view of the irradiation system with the X-ray tube (1). An ionizing chamber (2) was used for dose irradiation control measurements. (b) Top view of the position of four 25 cm[2] Flask bottles (3) to perform clonogenic assays with nanoparticles. The bottles were forming a square at the same distance from the center of the beam to have the same irradiation dose for all cells seeded.

5. Proton Irradiation

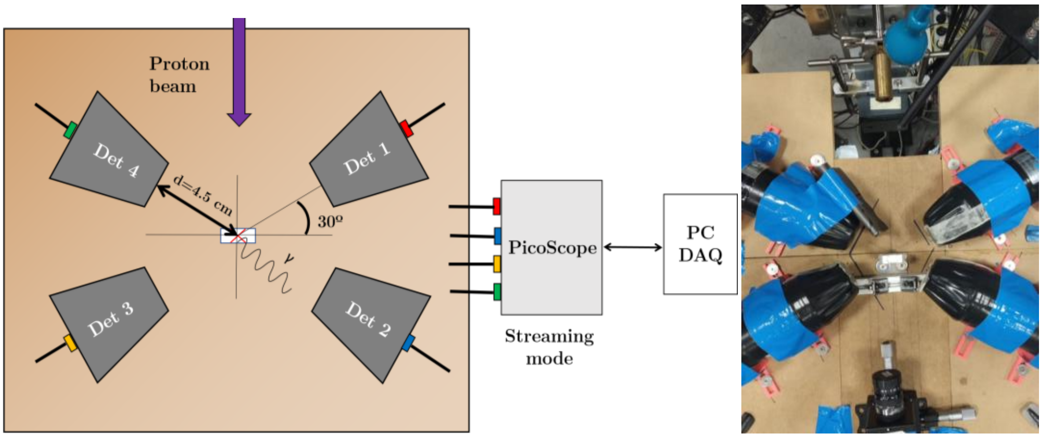

(a) (b)

Figure S4. (a) Scheme of the general view of the irradiation system with the proton beam and the four $LaBr_3(Ce)$-based detectors. (b) Top view of the real position of a sample in front of the beam and between the detectors.
